# Force-time metric responses to training in an Olympic 400m sprinter across a two-week microcycle: a case study

**DOI:** 10.3389/fspor.2026.1923398

**Published:** 2026-08-18

**Authors:** Dimitrije Cabarkapa, Amit Batra, Quincy R. Johnson, Damjana V. Cabarkapa, Alexander B. Wetmore, Thayne A. Munce

**Affiliations:** 1 Jayhawk Athletic Performance Laboratory – Wu Tsai Human Performance Alliance, Department of Health, Sport and Exercise Sciences, University of Kansas, Lawrence, KS, United States; 2 D2 Lab, Novi Sad, Serbia; 3 Faculty of Health Sciences and Physical Education, Kazimierz Wielki University, Bydgoszcz, Poland; 4 Sport Science Laboratory, Oklahoma State University, Stillwater, OK, United States; 5 Faculty of Physical Education and Sports Management, Singidunum University, Belgrade, Serbia; 6 Off The Mountain Strength, Park City, UT, United States

**Keywords:** fatigue, jump, neuromuscular, periodization, recovery, sprinting, training

## Abstract

Given the underrepresentation of female athletes in the scientific literature, this study investigated changes in neuromuscular performance across a two-week training microcycle in a world-class 400 m sprinter. The microcycle included resistance training, sprint speed, speed endurance, pace endurance, and resisted sprinting. Neuromuscular performance was assessed pre-post-practice using a countermovement vertical jump (CMJ) performed on a portable force plate system sampling at 1000 Hz (Hawkin Dynamics). Meaningful changes in performance were defined as values exceeding one standard deviation from baseline, established from 30 testing sessions conducted in the six months preceding the monitored microcycle. The results demonstrated clear acute post-practice alterations in neuromuscular function, with force and power-producing capacities declining in both the braking and propulsive phases of the CMJ immediately after practice, accompanied by reductions in jump height and reactive strength index-modified, particularly following sprint speed and endurance-based training sessions. These decrements were transient, with notable increases observed after rest days, suggesting a return toward or above the athlete's established baseline range. Overall, the observed performance fluctuations occurred alongside a periodized sequence of training and recovery days, illustrating the potential value of CMJ monitoring for tracking changes in performance across a training microcycle.

## 1 Introduction

The 400 m sprint is widely regarded as one of the most physiologically demanding and technically complex track and field events. Whereas shorter races such as the 100 m rely primarily on immediate phosphocreatine stores for energy, the 400 m requires athletes to maintain a near-maximal velocity for 45–60 seconds (1). Often described as a “prolonged sprint,” it merges the explosive power demands of short sprints with the endurance requirements typically seen in middle-distance events (2). This sustained effort places heavy reliance on glycolytic metabolism, while aerobic pathways also contribute to delaying fatigue and supporting performance (3–5). As a result, athletes experience substantial metabolic stress, with significant neuromuscular fatigue, muscle fiber disruption, and post-race blood lactate concentrations that can reach values as high as 19.1 mmol·L⁻^1^ (6).

Alongside the aforementioned metabolic constraints, the biomechanical and neuromuscular performance demands of the 400 m race further illustrate its complexity. Runners accelerate to peak velocity early in the race but inevitably experience substantial deceleration in the final 50 m (2, 7). This performance decline is strongly influenced by reductions in stride length and frequency, prolonged ground contact times, and altered muscle coordination patterns, particularly in hip extensors such as the gluteus maximus and biceps femoris (7). Historical analyses of major championship races confirm that the ability to preserve technical efficiency (250–400 m) under extreme fatigue is a distinguishing feature of world-class performers (2). Moreover, world-class 400 m sprinters were shown to display greater force-application capacity and superior stride mechanics compared to less experienced runners, allowing them to maintain longer strides, greater step frequencies, and ultimately faster overall race times (3, 8). Taken together, these findings demonstrate that success in the 400 m sprint depends on the integration of energy system capacity, neuromuscular resilience, and refined technical proficiency under conditions of extreme fatigue, reinforcing its status as one of the most physiologically demanding track and field events. Both sprint propulsion and the upward phase of the countermovement vertical jump (CMJ) require rapid and coordinated extension of the hip, knee, and ankle joints to generate force against the ground; therefore, CMJ force-time characteristics may serve as a practical surrogate for assessing integrated lower-body neuromuscular fatigue relevant to sprint performance (8, 12, 13).

Neuromuscular function plays a central role in sustaining performance and tolerating fatigue, which has led to its increased focus in athlete monitoring practices (9–11). A commonly used, non-invasive, and time-efficient testing modality for this purpose is the CMJ performed on force plates. While jump height is the most frequently reported outcome, CMJ assessments provide a much broader profile of neuromuscular status through force-time variables such as peak force and power within both the braking (i.e., eccentric) and propulsive (i.e., concentric) phases of the jumping movement (12–14). These measures have shown to be sensitive to both acute fatigue and chronic training adaptations, giving practitioners a more detailed understanding of athlete readiness and recovery status (15, 16). For example, Gathercole et al. (12) reported reductions in eighteen neuromuscular performance variables immediately following a fatiguing high-intensity protocol in collegiate team-sport athletes, with most measures trending back toward baseline within 24 hours post-exercise. Furthermore, recent scientific evidence demonstrates that meaningful changes in CMJ force-time metrics can occur even when jump height remains unchanged, indicating that underlying neuromuscular alterations may precede observable performance decrements (17). Still, despite the widespread application of CMJ testing, limited research has investigated longitudinal responses in world-class sprinters, with an even greater lack of data available for female athletes across multiple training sessions within a structured microcycle. Although sex-related physiological characteristics may potentially influence neuromuscular fatigue and recovery, these responses have not been sufficiently characterized in world-class 400 m sprinters. Consequently, female-specific longitudinal data are needed before conclusions can be drawn regarding whether training-induced fatigue and recovery profiles are meaningfully sex dependent.

Therefore, the purpose of this case study was to monitor changes in neuromuscular performance parameters across a two-week training microcycle, as well as pre-post-practice changes, in a female Olympic-level 400 m sprinter. Based on the currently available scientific literature, it was hypothesized that multiple force-time metrics (e.g., RSI-modified, jump height, peak braking and propulsive force and power) would be altered in response to an acute bout of exercise (e.g., sprint training, pace endurance, resistance exercise), even when accounting for the athlete's well-trained status.

## 2 Materials and methods

### 2.1 Participants

An Olympic-level female athlete specializing in the 400 m sprint (*n* = 1) volunteered to participate in this case study. During the testing period, the athlete maintained full participation in coach-prescribed training regimens and reported no musculoskeletal injuries that could compromise sprinting or jumping performance. Based on the participation classification framework proposed by McKay et al. (18), this athlete can be classified at a Tier 5 (i.e., world-class) athlete, representing the highest level of training and performance caliber. Due to high performance level, world-class competitive success, and willingness to participate in repeated testing procedures, the athlete was purposively selected for this individualized case study. All testing procedures were approved by the University's Institutional Review Board, and the athlete provided written informed consent prior to participation.

### 2.2 Training plan

The athlete's training program was designed and supervised by national team coaching staff who had been working with the athlete for more than two years. The two-week microcycle incorporated multiple training modes, including: (i) resistance training (i.e., weight room), (ii) sprint speed, (iii) speed endurance, (iv) pace endurance, and (v) resisted sprinting. Resistance training, resisted sprinting, sprint-speed training, and speed-endurance training were each performed once per week. Pace-endurance training was performed twice during the first week and once during the second week. The sequence of training sessions was prescribed by the coaching staff as part of the athlete's established periodized program and was not altered for research purposes; therefore, pre-measurements reflected the athlete's condition at the beginning of each session, including any potential accumulated effects of preceding training.

The resistance training sessions consisted of both lower-body and upper-body multi-joint exercises, followed by core (i.e., abdominal and back) exercises. The lower-body exercises included back squats, half squats, cleans from the knee, Bulgarian split squats, hip thrusts, and single-leg deadlifts. The upper-body exercises included bench press, pull-ups, and single-arm dumbbell rows. Training intensity during the microcycle was prescribed at 80–95% of one-repetition maximum. The athlete typically performed 3–8 repetitions per exercise across 3–5 sets, with approximately two-minute rest intervals between each set. In addition, each resistance training session included mobility exercises and jumping and plyometric movements such as hurdle jumps, alternate bounds, shot put throws, etc.

Sprint speed training consisted of 40 and 60 m sprints, including block starts. The target intensity was 95–100% of maximal effort, with 3–5 minutes of rest between each sprint trial. The total running volume per session was kept below 300 m. Speed endurance training consisted of runs ranging from 80 to 150 m at intensities exceeding 95% of maximal effort. Rest intervals between repetitions were 5–8 minutes, and the total running volume per session was kept below 1200 m. Pace endurance training sessions consisted of runs ranging from 300 to 500 m. The prescribed intensity was 80–95% of maximal effort, with rest intervals of 2–8 minutes. The total running volume for pace endurance training sessions ranged between 2500 and 3500 m. Lastly, resisted sprinting training sessions were performed using the 1080 Motion (Stockholm, Sweden), a computer-controlled motorized device that allows precise application of resistance or assistance during sprinting, while simultaneously capturing real-time performance data. The applied load was set between 1–3 kg, with rest intervals of 3–5 minutes between each sprint trial, and the total running volume per resisted sprinting training session was maintained between 300 and 600 m.

### 2.3 Testing procedures

To assess lower-body neuromuscular performance, the athlete performed a CMJ on a uni-axial force plate system (Hawkin Dynamics, Model 0484; Westbrook, ME, USA) sampling at 1000 Hz. All CMJs were executed without an arm swing (i.e., hands placed on the hips throughout the movement). Strong verbal encouragement was provided to ensure maximal effort, and athlete were instructed to focus on applying force against the ground as explosively as possible. Each jump was separated by a 10-second rest interval, and the mean value across three trials was used for subsequent performance analysis.

The testing procedures were conducted on a pre-post-practice basis. Prior to each practice session, the athlete completed a standardized raise, activate, mobilize, and potentiate (RAMP) warm-up protocol (15 min) administered by the athlete's strength and conditioning practitioner to ensure standardization of testing procedures. The warm-up represented a standardized, low-intensity routine habitually performed by the athlete and was well below her typical training demands. The same protocol was completed before every pre-assessment to ensure consistency across testing sessions. Post-practice CMJ testing was conducted within five minutes of completing each training session, except after high-intensity pace-endurance and resisted-sprinting sessions, when testing was deemed inappropriate because of the athlete's substantial acute training exposure. Following CMJ testing, ten force-time metrics of interest were analyzed: braking phase duration, propulsive phase duration, time-to-takeoff, countermovement depth, reactive strength index (RSI)-modified (i.e., jump height divided by contraction time) and peak braking and propulsive force and power. The validity and reliability of the aforementioned metrics have been previously documented in the scientific literature (19–21). A detailed list and definitions of each dependent variable examined in this study are listed in Table 1.

**Table 1:** List of dependent variables examined in the present study and their definitions.

Variable (unit)	Definition
Braking phase duration (msec)	The time taken to complete the braking phase.
Propulsive phase duration (msec)	The time taken to complete the propulsion phase.
Peak braking force (N)	The peak force applied to the system COM during the braking phase.
Peak propulsive force (N)	The peak force applied to the system COM during the propulsion phase.
Peak braking power (W)	The peak power applied to the system COM during the braking phase.
Peak propulsive power (W)	The peak power applied to the system COM during the propulsion phase.
Jump height (m)	Maximum jump height calculated via impulse-momentum calculation.
Countermovement depth (m)	The peak negative vertical displacement of the system COM.
RSI-modified (ratio)	Jump height divided by contraction time.
Time-to-takeoff (msec)	The total time taken from the initiation of the movement to takeoff.

COM, center of mass; RSI, reactive strength index.

The athlete regularly performed CMJ testing procedures to monitor readiness status and to track changes in neuromuscular performance as part of the training regimen administered by the coaching staff. Baseline values (x¯ ± SD) for each of the aforementioned force-time metrics were established by averaging results from 30 testing sessions conducted over the six months prior to the evaluated microcycle. These baseline sessions were performed when the athlete was well-rested to ensure that values were not influenced by acute or chronic fatigue. The resulting baseline values for the ten force-time metrics of interest served as anchor points (i.e., criterion measures) for evaluating performance changes during the current testing period.

In addition to CMJ testing, finger-stick blood lactate samples were collected using a Lactate Pro device with single-use disposable lancets (Arkray, Kyoto, Japan), which has been shown to demonstrate strong intra-device reliability (22). As commonly implemented during pace endurance training, blood lactate measurements were obtained to assess the athlete's glycolytic contribution, lactate tolerance, buffering capacity, and training adaptations, all of which are key determinants of 400 m sprint performance. To capture the physiological response, blood lactate concentrations were measured at both the third and twentieth minute of the pace endurance training session.

Complementary to these physiological measures, rating of perceived exertion (RPE) was assessed as a subjective indicator of internal load immediately after each training session using the CR-10 scale (23, 24). Session RPE training load was then calculated by multiplying the reported RPE by the training duration (min). Unlike blood lactate measurements, which were only collected during the pace endurance training session due to their invasive nature and time constraints, RPE and session training load were obtained for every training session. This approach provided a consistent non-invasive measure of internal load across the entire microcycle, allowing for daily monitoring of the athlete's perceived exertion and overall training demands (25).

### 2.4 Statistical analysis

Sports science research often relies on group-based statistics, which yield generalizable findings but may overlook individual responses (26–29). However, for elite athletes, single-subject case study designs (*n* = 1) may be more appropriate. These designs are frequently limited by the absence of controlled conditions, small sample sizes, and the dynamic nature of high-performance environments, resulting in a quasi-experimental approach.

As proposed by Chavda (26), this study adopted an individualized analytical framework to identify meaningful deviations in neuromuscular performance characteristics across a two-week microcycle in a 400 m sprinter. The objective was not to generalize findings to a wider population, but to capture subtle performance variations in a single Olympic-level athlete. To balance sensitivity to individual performance fluctuations with the potential for measurement variability, performance outcomes were interpreted using individualized one-standard-deviation (1SD) thresholds. Accordingly, all force-time metrics were reported as mean ± standard deviation (x¯ ± SD). Baseline values were calculated from 30 testing sessions conducted during the six months preceding the monitored microcycle. The mean 1SD range derived from these sessions represented the athlete's established baseline performance range for each force-time metric. Each pre-post-practice observation was independently compared with the corresponding individualized baseline boundaries. Values falling below the lower 1SD boundary were flagged as low, values exceeding the upper 1SD boundary were flagged as high, and values between the two boundaries were classified as being within the athlete's baseline range. These classifications represented deviations from the athlete's established performance profile and were not based on whether the pre-post difference exceeded 1SD.

## 3 Results

**Figure 1:**
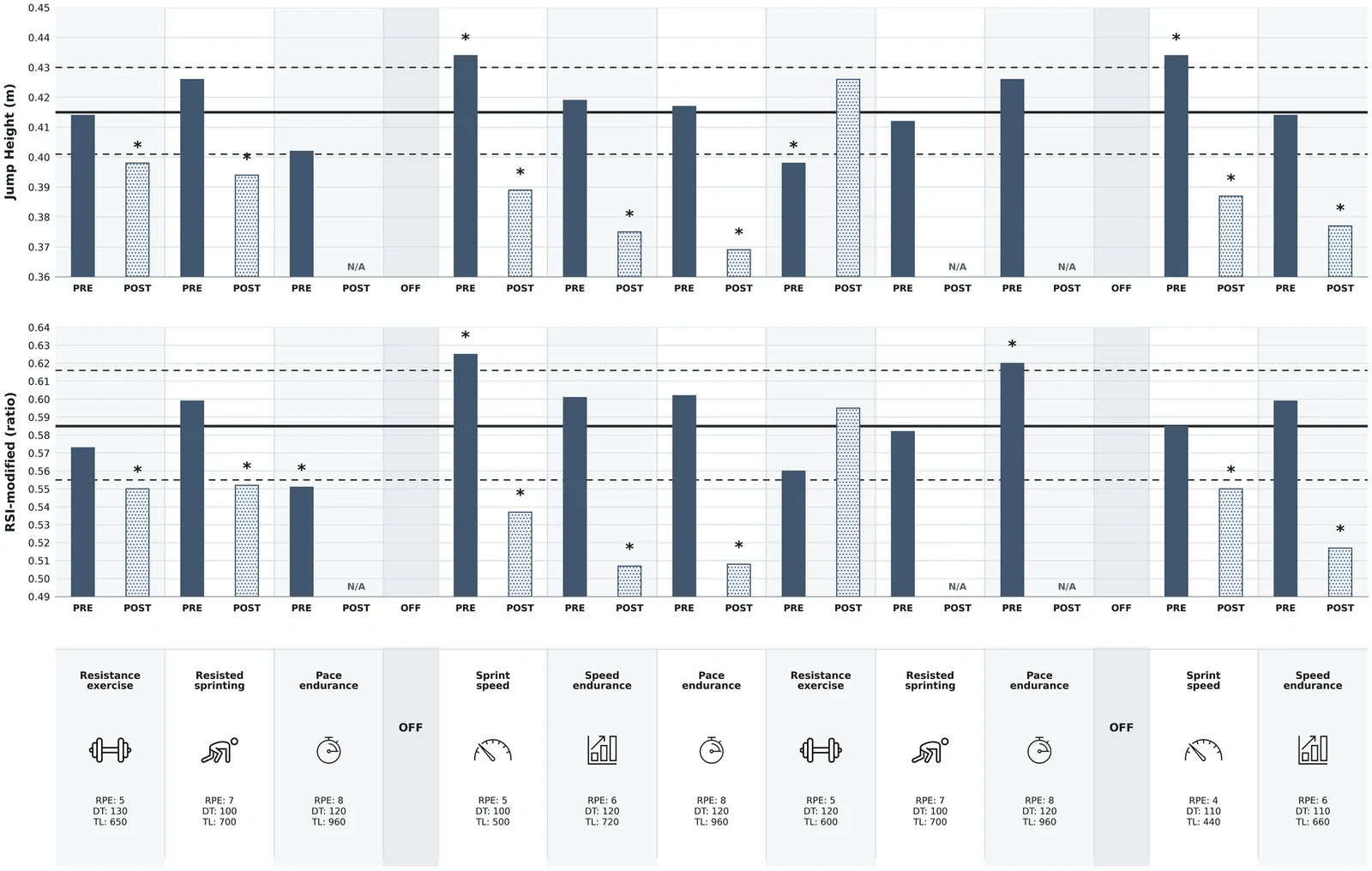
Changes in countermovement vertical jump height and reactive strength index (RSI)-modified (i.e., outcome metrics) across a two-week microcycle. RPE, rating of perceived exertion; DT, duration; TL, training load; N/A, not available post-training data. The bold horizontal line represents the baseline value for each performance metric. The dashed horizontal lines indicate the upper and lower one-standard-deviation boundaries derived from the established baseline. (*) – notable change in the variable outside of the baseline range. Solid bars represent pre-training values, whereas shaded bars represent post-training values.

**Figure 2:**
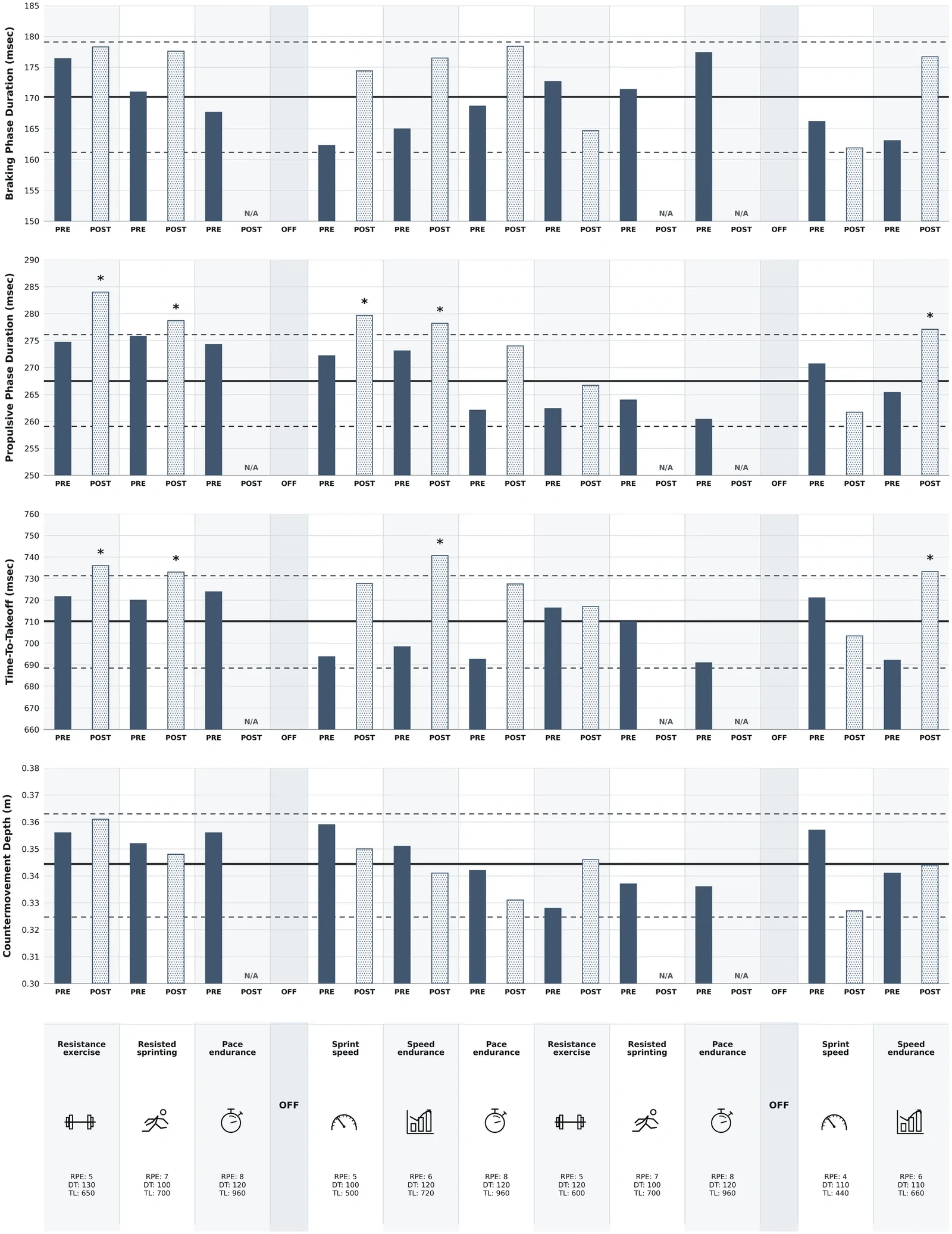
Changes in countermovement vertical jump strategy metrics across a two-week microcycle. RPE, rating of perceived exertion; DT, duration; TL, training load; N/A, not available post-training data. The bold horizontal line represents the baseline value for each performance metric. The dashed horizontal lines indicate the upper and lower one-standard-deviation boundaries derived from the established baseline. (*) – notable change in the variable outside of the baseline range. Solid bars represent pre-training values, whereas shaded bars represent post-training values.

**Figure 3:**
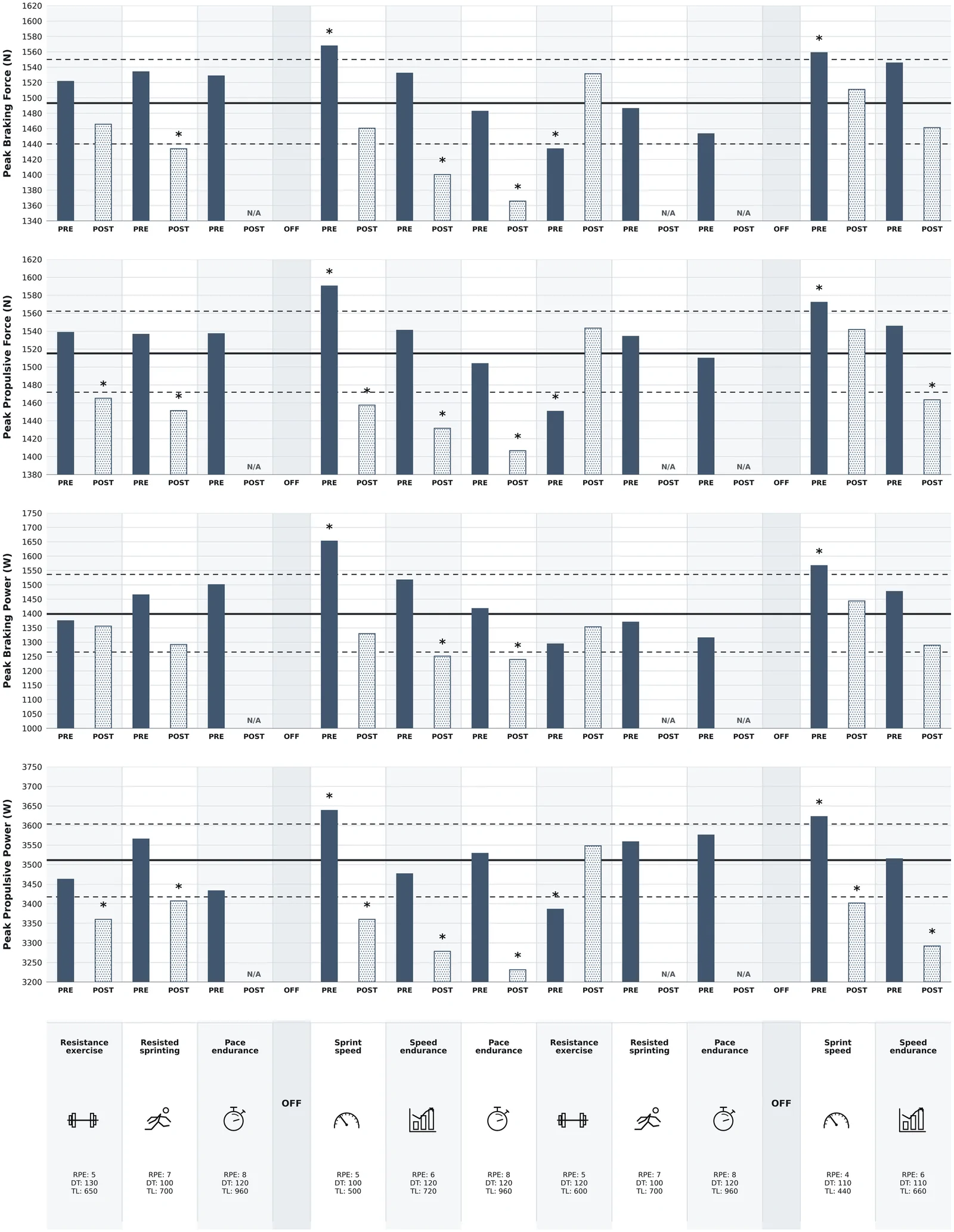
Changes in countermovement vertical jump force and power metrics across a two-week microcycle. RPE, rating of perceived exertion; DT, duration; TL, training load; N/A, not available post-training data. The bold horizontal line represents the baseline value for each performance metric. The dashed horizontal lines indicate the upper and lower one-standard-deviation boundaries derived from the established baseline. (*) – notable change in the variable outside of the baseline range. Solid bars represent pre-training values, whereas shaded bars represent post-training values.

Descriptive statistics, mean and standard deviation (x¯ ± SD), for each force-time metric of interest are presented in Table 2, providing the baseline athlete profile (i.e., criterion measure) against which all subsequent testing changes were compared. The detailed session-by-session data, including the fluctuations pre-post practice for each force-time metric, RPE, duration and training load are presented in Figures 1–3.

**Table 2:** Countermovement vertical jump neuromuscular profile of an Olympic 400 m sprinter.

Variable (unit)	Value
Braking phase duration (msec)	170.1 ± 8.9
Propulsive phase duration (msec)	267.6 ± 8.8
Peak braking force (N)	1495.2 ± 54.2
Peak propulsive force (N)	1517.3 ± 44.4
Peak braking power (W)	1396.6 ± 143.5
Peak propulsive power (W)	3510.6 ± 96.2
Jump height (m)	0.415 ± 0.016
Countermovement depth (m)	0.344 ± 0.019
RSI-modified (ratio)	0.586 ± 0.031
Time-to-takeoff (msec)	710.0 ± 21.1

RSI, reactive strength index.

For the outcome metrics, post-practice jump height fell below the lower 1SD baseline boundary following the resistance exercise and resisted sprinting sessions during the first week, both sprint-speed sessions, both speed-endurance sessions, and the second pace-endurance session during the first week. RSI-modified showed a similar post-practice pattern, falling below the lower boundary following the same sessions. In addition, pre-practice RSI-modified was below the lower boundary before the first pace-endurance session. In contrast, pre-practice jump height exceeded the upper 1SD boundary before both sprint-speed sessions, whereas RSI-modified exceeded the upper boundary before the sprint-speed session during the first week and the pace-endurance session during the second week. During the resistance exercise session in the second week, jump height increased from below the lower boundary before practice to within the baseline range after practice.

For the strategy metrics, braking phase duration and countermovement depth remained within the individualized 1SD baseline boundaries throughout the two-week microcycle. Conversely, propulsive phase duration exceeded the upper boundary after the resistance exercise, resisted-sprinting, sprint-speed, and speed-endurance sessions during the first week, as well as after the speed-endurance session during the second week of training. Time-to-takeoff exceeded the upper boundary after the resistance exercise and resisted-sprinting sessions during the first week and after both speed-endurance sessions. Thus, the meaningful increases in time-to-takeoff were primarily associated with a lengthening of the propulsive phase, whereas braking phase duration remained within the baseline boundaries.

Force and power-related metrics also demonstrated session-specific changes. Before both sprint-speed sessions, peak braking force, peak propulsive force, peak braking power, and peak propulsive power exceeded their respective upper 1SD boundaries. Post-practice peak propulsive power fell below the lower boundary after the resistance exercise, resisted sprinting, sprint-speed, speed-endurance, and second pace-endurance sessions during the first week, as well as after the sprint-speed and speed-endurance sessions during the second week of training. Peak propulsive force fell below the lower boundary after the resistance exercise, resisted sprinting, sprint-speed, speed-endurance, and second pace-endurance sessions during the first week, as well as after the speed-endurance session during the second week. Peak braking force fell below the lower boundary after the resisted-sprinting, speed-endurance, and second pace-endurance sessions during the first week, whereas peak braking power fell below the lower boundary after the speed-endurance and second pace-endurance sessions during the first week. Before the resistance exercise session during the second week, peak braking force, peak propulsive force, and peak propulsive power were below their respective lower boundaries but returned to within the baseline range after practice.

In addition, blood lactate concentrations for the first, second, and third pace-endurance sessions were 17.3 and 15.2, 17.7 and 15.2, and 16.1 and 14.2 mmol·L⁻^1^ at the three-minute and twenty-minute time marks, respectively.

## 4 Discussion

The purpose of the present study was to monitor changes in neuromuscular performance characteristics across a two-week training microcycle, as well as examine their pre-post-practice fluctuations, in a female Olympic-level 400 m sprinter. Given that female athletes remain underrepresented in the scientific literature compared with their male counterparts, this study helps to address a critical gap in current knowledge. The results revealed clear acute post-practice alterations in lower-body neuromuscular function. Specifically, force and power-producing capacities tend to decrease in both the braking and propulsive phases of the CMJ immediately after practice, whereas notable increases were observed following off-days (i.e., rest period), suggesting effective post-exercise recovery. Also, RSI-modified and jump height exhibited a similar pattern, with substantial reductions post-practice, particularly after sprint speed, speed endurance, and pace endurance training sessions. In contrast, strategy-related metrics such as braking-phase duration and countermovement depth remained stable across the two-week microcycle, whereas propulsive phase duration was prolonged post-practice relative to pre-practice values. As a result, overall time-to-takeoff increased, driven primarily by lengthening of the propulsive phase rather than changes in braking phase duration. Collectively, these findings advance the understanding of acute CMJ-derived performance responses to training in a world-class female athlete and provide practical implications for monitoring strategies and resistance training practices in high-performance environments.

The development and maintenance of physiological characteristics that underpin peak athletic performance have long been a central priority for sports performance professionals and have been studied extensively. Historical records from ancient Rome describe military training methods that followed an organized and sequential structure, targeting the cardiovascular and musculoskeletal systems, general combative skills, specific tactical abilities, and integrated training strategies designed to enhance dynamic correspondence (i.e., the transfer of training) (30). Over time, and with advances in sport science, many of these foundational approaches have endured and been refined, ultimately shaping contemporary training methods for modern athletes (31–33). Building on this historical foundation, the findings of the present study emphasize the importance of structured and sequential periodization of training loads, programming exercises that effectively modulate fatigue to optimize long-term adaptations and implementing athlete monitoring strategies to maximize preparedness for peak performance. While periodization refers to the macro-management of the training process across longer timeframes (e.g., macro, meso, and microcycles), programming reflects the micromanagement of smaller training units such as individual workout sessions (32). At the microcycle level, typically spanning several days to a week, this athlete was prescribed a sequenced progression characterized first by gradual loading and accumulation, followed by training focused on the transmutation of sprint speed and speed endurance, two critical performance determinants of the 400 m sprinting event.

Although higher training volumes and intensities coincided with reductions in several CMJ metrics during the earlier stages of the microcycle, values for several metrics increased during the latter stages of the microcycle. These observations may reflect changes in readiness or recovery status; however, physiological adaptation was not directly assessed. The sequenced progression from resistance exercise to sprint speed development, and subsequently to sprint endurance training at the microcycle level, highlights the precision required in exercise selection, ordering, and progression for optimizing physical qualities in world-class athletes. These approaches are consistent with prior research reports emphasizing the benefits of structured and sequenced training regimens for athletic populations (31–36), the downstream biological adaptations associated with resistance exercise (37), as well as the influence of biorhythms and exercise chronobiology on fitness, fatigue, and recovery (38, 39). Furthermore, they directly align with established training principles for top-tier athletes (34, 35, 40–42). Thus, the findings of the present study also address a critical gap in the scientific literature by providing novel insights into training periodization and preparation strategies for world-class female athletes.

When designing training programs, classic variables such as load, frequency, duration, and intensity can be manipulated to support optimal athlete preparation, performance, and progressive overload (43). More recently, greater emphasis has been placed on exercise selection, order, and rest intervals as critical factors in resistance training biology and program design methodologies (37). Within the present study, key observations also emerged regarding the manipulation of program variables. At a general level, training appeared to follow a three-session microcyclic sequence: resistance exercise in the first session, resisted sprinting in the second, and pace endurance in the third. Conceptually, this progression reflects a logical transition from developing and maintaining neuromuscular force, a general quality of athletic populations, to applying that force in a more specific task such as resisted sprinting, and ultimately to sport-specific demands emphasizing endurance at relevant durations and intensities. Overall, the findings of this case study reinforce the importance of holistic athlete development, even within the context of single-event specialization, and align with previous work in track and field athletes (34). For example, Anousaki et al. (34) observed a 25-week periodized training macrocycle that focused on developing muscular strength, power, and the translation of each prior to a national outdoor championship within a cohort of well-trained track and field throwers who trained up to 10 times per week. At the mesocyclic level, two primary periods of training were identified: i) hypertrophy and maximum strength, and ii) maximum strength and power, lasting 11 and 10 weeks, respectively. Within each mesocycle, microcycles lasting between one and three weeks were observed. These microcycles progressively increased in intensity (i.e., accumulation phase) while decreasing in volume (i.e., realization phase), ultimately leading to a final transmutation phase focused on power and speed development ahead of competition. Observations from the current study related to program design variables, such as RPE, session duration, and training load, align with this model, showing a gradual increase in intensity and volume prior to transitioning into a new period of training emphasis.

Besides the aforementioned trends that observed across the periodized training, pre-post-practice fluctuations in neuromuscular performance have been consistently observed in the scientific literature across both individual and team sport athletes (44–47). For instance, Cabarkapa et al. (44) reported notable decreases in peak propulsive power, jump height, and RSI-modified, along with increases in propulsive phase duration and time-to-takeoff following a two-hour basketball practice session in professional players. These responses were directionally consistent with the observations made in the present study, where force and power-producing capacities decreased in both the braking and propulsive phases of the CMJ, alongside reductions in jump height and RSI-modified. However, the mechanical and metabolic demands of the two sports differ considerably. The 400 m sprint is characterized predominantly by high-velocity linear running and substantial glycolytic stress, whereas basketball involves intermittent multidirectional movements, repeated jumping, and frequent accelerations and decelerations (4, 5, 25, 48). Therefore, although similar CMJ alterations may occur, the underlying sources of fatigue and subsequent recovery profiles may differ between these athletic populations. As no direct cross-sport comparison was performed, these differences should be interpreted cautiously.

Similarly, Gathercole et al. (46) demonstrated that immediately after a fatiguing running protocol, widespread declines in lower-body CMJ performance parameters occurred, including reductions in propulsive force, mean and peak power, propulsive phase duration, and jump height. However, changes in CMJ force-time metrics should not be interpreted as direct evidence of central or peripheral neuromuscular fatigue. Lindsay and Fletcher (49) observed that prolonged low-frequency force depression remained present for up to 60 min following exhaustive, metabolically demanding cycling exercise, whereas only concentric impulse was reduced during the first eight minutes of recovery and the remaining CMJ variables were unchanged. These findings indicate that CMJ performance may be dissociated from directly assessed peripheral neuromuscular fatigue, particularly following metabolically demanding exercise with minimal eccentric loading (49). In the present study, CMJ testing was conducted approximately five minutes after training and may therefore have captured transient alterations in force-production capacity. Nevertheless, because contractile responses and markers of muscle damage were not assessed, the physiological mechanisms underlying the observed CMJ changes cannot be fully determined. Accordingly, the present findings should be interpreted as acute alterations in CMJ-derived performance rather than direct confirmation of neuromuscular fatigue, prolonged low-frequency force depression, or exercise-induced muscle damage.

While the present study provides novel insights into neuromuscular performance fluctuations in a world-class female athlete, several limitations should be acknowledged. As a single-subject case study (*n* = 1), the findings cannot be generalized to broader athletic populations and should be interpreted within the context of individualized performance monitoring strategies. Moreover, the analysis was primarily limited to neuromuscular performance characteristics, without the detailed concurrent monitoring of external load (e.g., running distance) or internal load (e.g., heart rate, physiological biomarkers), as well as menstrual cycle, which would offer a more comprehensive evaluation of training stress and adaptation. Also, the two-week observation period may not fully capture long-term training responses or variability across different mesocycles and competitive phases. Thus, future research should employ larger and more diverse samples, extended monitoring durations, and integrated assessments of neuromuscular, external, and internal load measures to enhance the ecological validity and translational relevance of such findings.

## 5 Conclusion

In summary, this case study demonstrated clear acute post-practice alterations in lower-body neuromuscular function in a world-class female 400 m sprinter across a two-week training microcycle. Both force and power-producing capacities declined in the braking and propulsive phases of the CMJ immediately after practice, accompanied by reductions in jump height and RSI-modified, particularly following sprint speed, speed endurance, and pace endurance sessions. Importantly, these decrements were transient, with notable increases observed after rest days, suggesting a return toward or above the athlete's established baseline range. The observed fluctuations occurred across a periodized sequence of training and recovery days, during which progressive loading was interspersed with recovery opportunities. Overall, these findings highlight the potential utility of integrating CMJ-based monitoring with periodized programming to inform training decisions and monitor readiness in world-class athletes.

## Data Availability

All relevant data is contained within the article.
